# Prevalence and risk factors associated with severe pre-eclampsia among postpartum women in Zanzibar: a cross-sectional study

**DOI:** 10.1186/s12889-020-09384-z

**Published:** 2020-09-04

**Authors:** Mwashamba M. Machano, Angelina A. Joho

**Affiliations:** Department of Nursing and Midwifery, College of Health Sciences, University of Dodoma, Dodoma, Tanzania

**Keywords:** Severe pre-eclampsia, Hypertensive disorders of pregnancy, Risk factors, Maternal mortality, Postpartum women, Gestational hypertension, East Africa, Tanzania, Zanzibar

## Abstract

**Background:**

Severe pre-eclampsia is more dominant in low and middle-income countries. In Sub-Saharan Africa, severe pre-eclampsia remains a major public health problem contributing to high rates of maternal mortality. Few studies have investigated the relationship between severe pre-eclampsia and associated factors in East Africa. The aim of this study was to determine the prevalence and risk factors associated with severe pre-eclampsia among postpartum women in Zanzibar.

**Methods:**

A hospital based analytical cross-sectional study design was used. Purposive sampling was utilized for the selection of hospitals. Proportionate sampling was used for selection of representatives from each hospital and participants were selected using systematic random sampling. Postpartum mothers were included in the study. The study was conducted by an interviewer who administered a questionnaire with close ended questions and chart review for data gathering. SPSS version 23 was used for data analysis and descriptive and multiple logistic regression was performed for control of confounders.

**Results:**

This study included a total of 400 participants with a 100% response rate. Participants ranged from 17 to 45 years of age with mean age (SD) of 28.78 (±6.296). The prevalence of severe pre-eclampsia among postpartum women was 26.3% (*n* = 105). After adjusting for the possible confounders, factors associated with severe pre-eclampsia were; maternal age group of 15–20 years (AOR 3.839; 95% C. I 1.037–14.210), pregnancy from new partner/husband (AOR 7.561; 95% C. I 3.883–14.724), family history of high blood pressure (AOR 6.446; C. I 3.217–12.917), diabetes prior to conception (AOR 55.827; 95% C. I 5.061–615.868), having high blood pressure in a previous pregnancy (AOR 19.382; 95% C. I 4.617–81.364), paternal age above 45 (AOR 2.401; 95% C. I 1.044–5.519) and multifetal gestation (AOR 7.62; 95% CI 2.01–28.84).

**Conclusion:**

The prevalence of severe pre-eclampsia among postpartum women in Zanzibar is high. Common risk factors in this setting include maternal age of 15–20 years, pregnancy with a new partner, family history of high blood pressure, pre-existing diabetes prior to conception, a history of high blood pressure in previous pregnancy paternal age greater than 45 and multifetal gestation.

## Background

Hypertensive disorders in pregnancy are one of the leading causes of morbidity, long-term disability and death during pregnancy and postpartum and account for approximately 14% of all maternal deaths worldwide [1]. Hypertensive disorders of pregnancy include: chronic hypertension; gestational hypertension; pre-eclampsia with or without severe features; eclampsia and chronic hypertension with superimposed pre-eclampsia [2, 3]. Hypertensive disorders are the second leading cause of maternal mortality worldwide [3]. A useful framework for understanding causes of maternal deaths includes the three delays model; 1) delay in deciding to seek care, 2) delay in reaching a healthcare facility 3) delay in receiving appropriate and correct care at the healthcare facility [4]. A woman with an obstetric emergency may delay seeking health care services because she does not know the complications and risk factors in pregnancy; she might have history of bad experience of health care; financial implications. The woman who have decided early and timely to seek health care may find obstacles in reaching the health facility as transport is limited in may settings. Upon arrival, she may experience a delay in receiving appropriate care because the health facilities lacks materials and supplies for her care and/or care providers are not optimally trained [4]. Each of these three delays impacts the morbidity and mortality among women with obstetric emergencies, including hypertensive disorders of pregnancy.

Pre-eclampsia complicates 2–8% of pregnancies globally and in Africa and Asia 9% of maternal deaths are attributed to pre-eclampsia [5]. From a global perspective, most deaths due to hypertensive disorders of pregnancy occur in developing countries [1]. The World Health Organization (WHO) estimates the incidence of pre-eclampsia in developing countries seven times higher (2.8% of live births) compared to more developed countries (0.4%) [4]. Pre-eclampsia is a multisystemic disorder of pregnancy associated with new-onset hypertension, which occurs most often after 20 weeks of gestation and frequently near term [2] with the presence of proteinuria or, in its absence, of signs or symptoms indicative of target organ injury [4]. Pre-eclampsia is categorized as being with or without severe features [2].

High resource countries classify pre-eclampsia with severe features with specific criteria. This includes new-onset severe range blood pressures (sBP) ≥ 160 mmHg or diastolic BP (dBP) ≥ 110 mmHg with or without proteinuria. Specific laboratory findings are also present with severe features including thrombocytopenia (platelet count less than 100,000 × 10^9^/L), impaired liver function as indicated by abnormally elevated blood concentrations of liver enzymes (to twice the upper limit normal concentration), and severe persistent right upper quadrant or epigastric pain unresponsive to medication and not accounted for by alternative diagnoses, renal insufficiency (serum creatinine concentration more than 1.1 mg/dL or a doubling of the serum creatinine concentration in the absence of other renal disease) pulmonary edema, new-onset headache unresponsive to medication and not accounted for by alternative diagnoses, or visual disturbances [2, 6]. In high resource settings, this type of clear classification of pre-eclampsia is more feasible. In contrast, many low resources settings do not have lab testing available nor have functioning equipment for blood pressure measurement. This makes diagnosis of pre-eclampsia a particular challenge in such settings. Often in low resource settings, health providers rely on a combination of blood pressure elevation plus clinical findings to make a diagnosis of pre-eclampsia with severe features. Often pre-eclampsia is not diagnosed and pregnant women present emergently with eclamptic seizures. Given high maternal mortality rates in low resource settings, the impact of pre-eclampsia is significant. In Sub-Saharan Africa alone, pre-eclampsia remains a major public health problem as the reported the prevalence of pre-eclampsia ranges from 1.8 to 16.7% and contributes to high rates of maternal mortality [3].

The adverse effects of severe pre-eclampsia have been reported in the literature, for example a study conducted on characteristics and outcomes of patient with eclampsia and pre-eclampsia in a rural hospital in Tanzania demonstrated a significant impact on neonates. In this study, 27% of perinatal deaths occurred among women with severe pre-eclampsia. In addition, more than one-third of neonates had a birth weight of less than 2.5kgs and 86% had birth weight less than 1.5kgs [5]. Furthermore, the study demonstrated that 38% of low birth weight babies did not survive and that poor neonatal outcomes were associated with long durations between admission time and delivery [4, 5].

The World Health Organization on its recommendations for prevention and treatment of pre-eclampsia and eclampsia identified key risks of obesity, chronic hypertension, diabetes, nulliparity, adolescent pregnancy and conditions leading to hyper-placentation and large placentas (e.g. twin pregnancy), previous pre-eclampsia, renal disease, autoimmune disease and multiple pregnancies [7]. Furthermore, risk factors for pre-eclampsia have been widely reported in sub-Saharan Africa, example is a retrospective study conducted at Kilimanjaro Christian Medical Center on prevalence and risk factors of pre-eclapsia and eclampsia, the factors including maternal age (≥35) years, ≥12 years of schooling, unmarried, overweight, obesity, hypertension and anaemia [8]. Moreover, obesity was reported as a risk factor for severe pre-eclampsia among sub-Sahan Africa women immigrated to Europe [9]. More literature have reported age 40 years or older, a pregnancy interval of more than 10 years, family history of pre-eclampsia, BMI of 35 kg/m2 or more, gestational age at presentation and pre-existing vascular disease (NICE, 2019).

Despite various efforts taken by the Tanzanian government in Zanzibar, it has a high maternal mortality rate of 647/100,000 live births with hemorrhage and hypertensive disorders as the leading causes of direct maternal death in Zanzibar [10, 11]. In Zanzibar, a study conducted at Mnazi Mmoja Referral Hospital revealed hypertensive disorders in pregnancy as the most frequent complication in all pregnancies, whereby severe pre-eclampsia was reported in 25.8% of potential life-threatening conditions and 13.5% of maternal near-miss events. Furthermore, the study found that severe pre-eclampsia contribute about 21.4% of maternal deaths [10].

In this study, the definition of severe pre-eclampsia included having gestational hypertension with severe range of blood pressure systolic blood pressure >160 mmHg and diastolic >110 mmHg urine with protein (proteinuria), swelling of face and extremities or generalized edema, blurred vision, severe headache, severe persistent right upper quadrant or epigastric pain unresponsive to medication and not accounted for by alternative diagnosis, or pulmonary edema. Classifying pre-eclampsia is important for delivery timing, as delivery is the treatment for pre-eclampsia. This study therefore sought to assess the prevalence and risk factors of severe pre-eclampsia among postpartum women in Zanzibar.

## Methods

### Study area

This study was conducted in four hospitals in Zanzibar which included: Mnazi Mmoja Hospital (Unguja Urban District), Kivunge Hospital (Unguja north “A”), Abdulla Mzee Hospital (Mkoani District Pemba) and Chake Chake Hospital (Chake Chake District Pemba). Zanzibar is found approximately 25 miles from the East Africa coast and is part of the Republic of Tanzania. Zanzibar is composed of two main islands, Unguja and Pemba with total of 2654 km^2^. As of 2019, Zanzibar had a total population of 1,303,569 whereby males were 630,677 (48.4%) and females 672,892 (51.6%). Approximately one-half of the population lives in urban settings (46.3%) while 53.7% live rurally. Women of reproductive age (15–49 yrs) is 339,007 with one-half (169,007) living in rural areas and the remainder (170,000) in urban areas. Zanzibar has a fertility rate of 5.1 [7]. Zanzibar’s main industries are tourism and agriculture.

### Study design and approach

A hospital based analytical cross-sectional study with a quantitative approach was utilized to assess the prevalence and risk factors of severe pre-eclampsia using questionnaire from postpartum women.

### Study populations

All postpartum women who delivered at gestational age of 28 weeks and above at the selected hospitals during the study period were included. Those who were seriously ill and mentally ill during data collection were excluded from the study.

### Sample size and sampling procedure

A total sample size of 400 participants was achieved using the formula for quantitative studies [26], assuming 51% proportion of severe pre-eclampsia from a previous study in Ethopia at a 5% margin of error and 95% confidence interval [8, 9].

Four hospitals were purposively selected with consideration of high number of deliveries. These hospitals included Mnazi Mmoja, Kivunje, Abdalla Mzee and Chake Chake. Proportionate sampling was then used to select number of participants from each of the selected hospital. A systematic random sampling technique was used to select participants in each hospital. Postpartum women who were able to respond to questions were approached within 72 h of delivery.

#### Data collection technique and procedure

A structured questionnaire was used for data collection with close ended questions. This standardized questionnaire was adopted and modified from FIGO [2016] and Subki & Algethami [12]. The questionnaire consisted of 5 demographic questions, 13 questions focused on clinical features, and 15 questions about risk factors. Questionnaires were prepared in English and translated into Kiswahili, the local language. Before the actual data collection process, a pilot study was conducted with 40 postpartum women to test the tool’s ability to obtain needed information prior to data collection and to identify confusing or ambiguous questions. Ambiguous questions were reworked or removed. Women included in the pilot study were not included in the final study. An interviewer administered the structured questionnaire to collect data. Four research assistants experienced in midwifery were trained in data collection and the specific questionnaire used in this study. Pre-eclampsia was confirmed through documentary review of labor and birth record, in patient chart records and the use of severe features (gestational hypertension with severe range of blood pressure systolic blood pressure >160 mmHg and diastolic >110 mmHg urine with protein (proteinuria), swelling of face and extremities or generalized edema, blurred vision, severe headache, severe persistent right upper quadrant or epigastric pain unresponsive to medication and not accounted for by alternative diagnosis, or pulmonary edema).

#### Data analysis

Statistical Product and Service Solution (SPSS) software version 23 was utilized to analyze the collected data. Descriptive analysis was used for demographic data, while Chi square was performed to identify the variables which were associated with severe pre-eclampsia. A binary logistic regression analysis was done mainly for controlling the possible confounders and identifies significant predictors of severe pre-eclampsia.

## Results

### Demographic characteristics of study participants

This study included 400 participants with a 100% response rate (due to study design (analytical cross-sectional study), there was no follow up and the tool used was an interviewer administered structured questionnaire). The mean age (SD) of participants was 28.78 (±6.3.0). The oldest respondent was 45 and the youngest respondent was 17 years old. Just over one-half of study participants 202 (50.5%) had secondary education while 162 (40.5%) had only completed primary education. Only six of the study participants (1.5%) had completed education beyond the secondary level. Majority of respondents lived in urban settings in Zanzibar 213 (53.3%). The overwhelming majority of women in this study were married 388 (97.0%) and 83% (332) of women reported that they did not have formal employment outside of their homes (Table 1).

**Table 1:** Demographics characteristics of the respondents (N-400)

VARIABLE	FREQUENCY	%
Age (years)		
15–20	37	9.3
21–35	297	74.3
36 and above	66	16.5
Marital status		
Single	12	3
Married	388	97
Residence		
Urban	213	53.3
Rural	187	46.8
Level of education		
No formal education	30	7.5
Primary education	162	40.5
Secondary education	202	50.5
Higher education	6	1.5
Occupation		
Not employed outside of home	332	83
Self employed	57	14.3
Employed	11	2.8

### Prevalence of severe pre-eclampsia

**Fig. 1:**
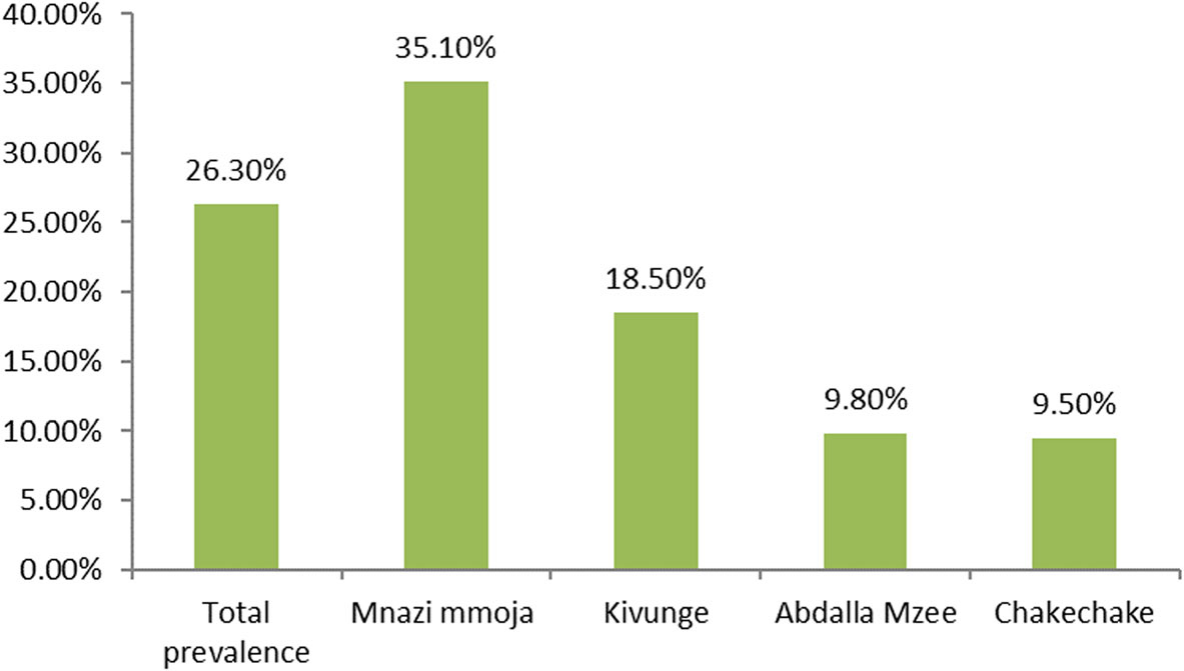
Prevalence of severe pre-eclampsia among postanal women

The total prevalence of severe pre-eclampsia among postnatal women in Zanzibar was 26.3% (105) but ranged depending on the facility. The prevalence of severe pre-eclampsia among admitted women at Mnazi Mmoja Hospital was 35.1%, whereby at Kivunge Hospital it was 18.5%, at Abdulla Mzee Hospital the prevalence was 9.8% and at Chake Chake Hospital was 9.5% (Fig. 1).

### Risk factors of severe pre-eclampsia among postnatal women in Zanzibar

Several factors were studied concerning risk factors associated with severe pre-eclampsia among postnatal women. In order to find the risk factors which had statistical relation with severe pre-eclampsia, a cross tabulation was done. Severe pre-eclampsia was significant associated with age (*P* < 0.01), residence (*P* = 0.02), pregnancy from new partner/husband (*P* < 0.00), pre-eclampsia in previous pregnancy (*P* < 0.00), diabetes prior to conception (*P* = 0.00), multifetal gestation (*P* = 0.00), family history of high blood pressure (*P* < 0.00), paternal age (*P* = 0.00) and age of last born (*P* = 0.00) (Table 2).

**Table 2:** Factors associated with severe pre-eclampsia (*N* = 400)

Variables	Severe pre-eclampsia		X^2^	*P*-value
	Yes	No		
	n (%)	n (%)		
Age (years)				
15–20	5 (13.5)	32 (86.5)		
21–35	74 (24.9)	223 (75.1)	9.26	0.01
36 and above	26 (39.4)	40 (60.6)		
Occupation				
House wives	89 (26.8)	283 (73.2)		
Self employed	13 (22.8)	44 (77.2)	0.41	0.82
Employed	3 (27.3)	8 (72.7)		
Marital status				
Single	2 (16.7)	10 (83.3)		
Married	103 (26.5)	285 (73.5)	0.59	0.44
Level of education				
No formal education	4 (13.3)	26 (86.7)		
Primary	49 (30.2)	113 (69.8)	4.31	0.23
Secondary	51 (25.2)	151 (74.8)		
Higher education	1 (16.7)	5 (83.3)		
Residence				
Urban	39 (20.9)	148 (79.1)		
Rural	66 (31)	147 (69)	5.28	0.02
Parity				
Primigravida	39 (26)	111 (74.4)		
Multipara	35 (25.2)	104 (74.8)	0.25	0.88
Grand multipara	31 (27.9)	80 (72.1)		
Pregnancy with a new partner				
Yes	51 (44.3)	64 (55.7)		
No	54 (18.9)	231 (81.1)	27.31	< 0.00
Pre-eclampsia in previous pregnancy				
Yes	7 (100)	0 (0)		
No	98 (24.9)	295 (75.1)	20.02	< 0.00
Diabetes prior to conception				
Yes	5 (83.3)	1 (16.7)	10.25	0.00
No	100 (25.4)	294 (74.6)		
High BP in previous pregnancy				
Yes	14 (82.4)	3 (17.6)		
No	91 (23.8)	292 (76.2)	28.87	< 0.00
Having multifetal gestation				
Yes	9 (56.3)	7 (43.7)		
No	96 (25)	288 (75)	7.75	0.00
High BP prior conception				
Yes	7 (87.5)	1 (12.5)		
No	98 (25)	294 (75)	15.82	< 0.00
Family history of high blood pressure				
Yes	43 (58.9)	30 (41.1)		
No	62 (19)	265 (81)	49.18	< 0.00
Household smoking				
Yes	16 (72.7)	6 (27.3)		
No	89 (23.5)	289 (76.5)	25.98	< 0.00
Paternal age				
< 45 yrs	79 (23.5)	257 (76.5)		
≥ 45 yrs	26 (40.6)	38 (59.4)	8.13	0.00
Number of abortions				
0–2	101 (29.5)	289 (74.1)		
3–10	4 (40)	6 (60)	1.00	0.32
Age of last born				
0–9	10 2 (25.7)	295 (74.3)		
10–15	3 (100)	0 (0)	8.49	0.00
ANC Visit				
< 4	57 (25.2)	169 (74.8)		
≥ 4	48 (27.6)	126 (72.4)	0.285	0.59

### Logistic regression analysis on risk factors associated with severe pre-eclampsia

In order to determine the risk factors associated with severe pre-eclampsia, binary logistic regression analysis was performed. Data analysis revealed that there was an association between the respondents being 15–20 years old and severe pre-eclampsia (OR = 4.16, *P* = 0.00). Other factors with apparent association included rural residence (OR = 1.70, *P* = 0.02), pregnancy with a new partner (OR = 3.41, *P* < 0.00), having pre-existing diabetes prior to conception (OR = 14.70, *P* = 0.02), having a history high blood pressure in a previous pregnancy (OR = 14.97, *P* < 0.00), multifetal gestation (OR = 3.86, P 0.01) and family history of high blood pressure (OR = 6.13, *P* < 0.00).

After adjusting for confounders, maternal age group of 15–20 years was four more times likely to develop severe pre-eclampsia when compared to those aged 35 years and above (AOR = 3.84; 95% C.I:1.04–14.21). Of note, rural women were 3 more times likely to develop severe pre-eclampsia when compared to urban (AOR =2.65; 95% C.I: 1.37–5.11). Those who were pregnant with a new partner were seven more times likely to develop severe pre-eclampsia compared to others (AOR = 7.56; 95% C.I: 3.88–14.72). Women with a family history of high blood pressure were 6.446 times more likely to develop severe pre-eclampsia compared to those who had no family history of high blood pressure. (AOR = 6.45; 95% C.I: 3.22–12.92) (Table 3).

**Table 3:** Multiple Logistic Regression for factors associated with severe pre-eclampsia (*N* = 400)

Variable			95% C. I				95% C. I	
	OR	*P*- value	Lower	Upper	AOR	*P* –value	Lower	Upper
Age								
15–20	4.16	0.01	1.44	12.06	3.84	0.04	1.04	14.21
21–35	1.96	0.02	1.12	3.43	1.92	0.11	0.86	4.31
36 and above	**(Ref)**							
Residence								
Urban	**(Ref)**							
Rural	1.70	0.02	1.08	2.69	2.65	0.00	1.37	5.11
Education level								
No formal	1.3	0.83	0.12	14.21	1.76	0.70	0.10	30.45
Primary	0.46	0.49	0.53	4.05	0.40	0.45	0.04	4.31
Secondary	0.64		0.68	5.19	0.45	0.51	0.04	4.87
Higher	**(Ref)**							
Pregnancy from new partner/husband								
No	**(Ref)**							
Yes	3.409	< 0.00	2.13	5.47	7.56	< 0.00	3.88	14.72
Diabetes prior conception								
No	**(Ref)**							
Yes	14.7	0.02	1.69	127.34	55.83	0.00	5.06	615.87
High blood pressure in previous pregnancy								
No	**(Ref)**							
Yes	14.97	< 0.00	4.21	53.27	19.38	< 0.00	4.62	81.36
Multifetal gestation								
No	**(Ref)**							
Yes	3.86	0.01	1.40	10.64	7.62	0.01	2.01	28.84
High blood pressure prior conception								
No	**(Ref)**							
Yes	21	0.01	2.55	172.82	10.45	0.06	0.90	121.57
Family member with high blood pressure								
No	**(Ref)**							
Yes	6.13	< 0.00	3.56	10.54	6.45	< 0.00	3.22	12.92
Household member smoking								
No	**(Ref)**							
Yes	8.66	< 0.00	3.28	22.72	10.41	< 0.00	3.03	35.76
Paternal age (years)								
< 45	**(Ref)**							
≥ 45	2.23	0.01	1.27	3.89	2.40	0.04	1.04	5.52

## Discussion

This study revealed a high prevalence of severe pre-eclampsia among postpartum women in Zanzibar at 26.3%. Since the current study was conducted in the referral hospitals, we might explore the antenatal pregnancy care currently available in these settings, to understand whether health care providers have the knowledge, skills and necessary clinical supplies to support early diagnosis of pre-eclampsia. This prevalence in the current study the rate is quite high when compared to other studies on pre-eclampsia in the global south which revealed a prevalence ranging from 1.8% to only 16.7% [13–15]. Our results are likely to be generalizable to the entire region, as our participating facilities were all referral sites from lower level facilities. However, we don’t know how long these mothers stayed at the primary health facilities before being transferred.

This high prevalence impacts the quality of life among pregnant women in Zanzibar and likely contributes to high levels of maternal mortality, morbidity and disability in this setting. One important impact of pre-eclampsia is that it increases the cost of health care services for families, communities and health systems as well increasing the number of hospitalizations and treatment cost (22). To reduce the impact of severe pre-eclampsia, health care workers must be equipped with knowledge and skills [19] in the management of this condition [29]. Moreover, they need to have supplies and medications such as magnesium sulfate for seizure treatment and prophylaxis and effective antihypertensive medications. Ideally, frontline health workers would be able to identify this condition at an early stage during antenatal clinic and provide adequate treatment and timely referral to prevent complications.

Governments continue to implement a range of strategies to reduce maternal and neonatal morbidity and mortality. These include innovative approaches to increase the use of skilled birth attendants to improve quality of care and increase maternal and newborn survival [16] and engaging community health care promoters to help with health education, mobilization and referral systems to help generate demand for services [17]. Furthermore, there is a need to strengthen community awareness among pregnant mothers and their partners on early signs of pre-eclampsia, the importance of early and regular ANC visits, and increase birth preparedness and complication readiness at the community level [17, 18]. To reduce maternal mortality and morbidity related to hypertensive disorders of pregnancy and other pregnancy related complications, community health care promoters can be effectively engaged at the community level to link women with the health systems. Well trained and supported community health promoters can provide essential health education at the community level and promote access to clinical services [17]. Empowering the community through training them on the early obstetric danger signs and importance of attending health care facilities will help to reduce the *first delay*. It will also increase their ANC visits which could support earlier detection, proper treatment and referral of women with obstetric emergencies to the higher-level management. Moreover, frontline nurses should have regular training in obstetric emergencies including severe pre-eclampsia and eclampsia for early identification, proper management and timely referrals of all mothers for the purpose of reducing complications and improving maternal and fetal survival [17]. There is a need for policy makers and leadership to insure the lab equipment and supplies are available within health facilities [19]. This will reduce the *third delay*.

Various studies have reported the use of acetylsalicylic (ASA) to help reducing the risk of pre-eclampsia and in high resource settings, and this is being rolled out routinely in many settings [19, 25, 30]. Given Zanzibar’s high rates of severe pre-eclampsia, perhaps warranting our exploration of the feasibility of the use of low dose ASA for the prevention of pre-eclampsia in this setting [19, 25, 30].

Our findings differ significantly from other studies looking at the diagnosis of pre-eclampsia with severe features during pregnancy. One study conducted previously in Zanzibar found the prevalence of severe pre-eclampsia at only 5%. That study used a sample size of 100 participants from only one hospital (Mnazi Mmoja Referral Hospital) and participants were all pregnant women [14]. Similarly, Kooffreh et al. at the University of Calabar found out that the prevalence of severe pre-eclampsia among pregnant women was only 1.2% in 2009 and 1.5% in 2010 [15], significantly lower than our findings. In contrast, our study utilized a larger sample size of 400 participants from four different hospitals and the study population was systematic randomly sampled postpartum women around the time of birth. If we were to follow women through the entire postpartum period, we would anticipate the number to rise further as pre-eclampsia can develop within the postpartum period as well. This approach was demonstrated in a study done by Zenebe et al. [9] in Ethiopia whereby the prevalence of severe pre-eclampsia was found to be 51.9%. This study was conducted in Ethiopia using a prospective cross-sectional design and was conducted for 1 year from April 2009 to March 2010. In contrast, our current study used only 1 month to collect data and focused on immediate postpartum women, rather than all pregnant and postpartum women. To best quantify the impact of hypertensive disorders in the peripartum period, a longer-term prospective study would be helpful.

Regardless, the current study in Zanzibar identified some clear patient risk factors in this setting that are strongly associated with severe pre-eclampsia. These mirror risk factors mirror those widely reported in the international literature. These include: history of high blood pressure in a previous pregnancy, having multiple pregnancy, history of chronic hypertension, paternal age above 45 years, and history of high blood pressure in the previous pregnant [20], having diabetes prior to conception [28], family history of high blood pressure [21, 31], pregnancy from new partner/husband [22], maternal age of 15–20 years [23] and family history of pre-eclampsia [24, 27, 31]. Since the current study was conducted in the referral hospitals, we might explore the antenatal pregnancy care currently available in these settings, to better understand whether health care providers have the knowledge, skills and necessary clinical supplies to support early diagnosis of hypertension disorders of pregnancy, including pre-eclampsia.

## Conclusion

Based on our findings, it is observed that the prevalence of severe pre-eclampsia among post-delivery women in Zanzibar is high. Common risk factors of severe pre-eclampsia included maternal age group of 15–20 years, being pregnant with a new partner, family history of high blood pressure, having diabetes prior conception, having a history of high blood pressure in previous pregnancy, having a multifetal gestation, having a diagnosis of chronic hypertension and paternal age of above 45 yrs.

The maternal mortality rate on Zanzibar is unacceptably high at 647 maternal deaths per 100,000 live births [10, 11]. Given that the prevalence of women who develop severe pre-eclampsia during pregnancy, birth and the postpartum period is also high in this setting, we have uncovered an urgent opportunity to improve maternal health care delivery and save lives. Risk factors have been well established in many international studies focused on pre-eclampsia and are also reflected in our study findings. To reduce direct and indirect maternal morbidity and mortality from hypertensive disorders in pregnancy, including pre-eclampsia and eclampsia – better care for women is required during all phases of their pregnancies, especially in the Global South. A multi-pronged approach is urgently needed in this setting to support communities and frontline health care providers to provide more equitable and evidence-based maternal health care. We have identified a clear and urgent need for some innovative approaches that can improve maternal and newborn survival. These include strategies that target community engagement for birth preparedness and complication readiness and ensuring that birth attendants working in health facilities are truly skilled and equipped.

Though our study was conducted carefully, it is not without limitations. First, our study was a cross sectional study we included postpartum women around the time of birth. If we were to follow women from ANT care through birth and the entire postpartum period (6 weeks), we would anticipate the number to rise further as severe pre-eclampsia can develop during antepartum, at birth and within the postpartum period as well. Therefore, there is a need of conducting the same study longer-term prospective study.

Second, there was a challenge in diagnosing severe pre-eclampsia with severe features in the health facilities included in this study, there was limitation of use of laboratory tests such as thrombocytopenia, liver and renal function tests. We therefore, encourage health facilities to use laboratory tests with severe features in diagnosing severe pre-eclampsia. Third, this study was conducted in the referral hospitals, we don’t know if these women delayed reaching the primary health facilities or there were delays in referring them.

## Data Availability

Data set is available upon request to the corresponding author.

## Abbreviations

ANC: Antenatal care
BMI: Body mass index
C.I: Confidence interval
FIGO: International federation of gynecology and obstetrics
NBS: National bureau of statistics
NICE: National institute for clinical excellence
OCGS: Office of chief government statistician
WHO: World health organization
